# Epithelial Tight Junctional Changes in Colorectal Cancer Tissues

**DOI:** 10.1100/tsw.2011.86

**Published:** 2011-04-05

**Authors:** Xuexuan Wang, Owen Tully, Benjamin Ngo, Marc Zitin, James M. Mullin

**Affiliations:** ^1^ Lankenau Institute for Medical Research, Wynnewood, PA, USA; ^2^ Division of Gastroenterology, Lankenau Medical Center, Wynnewood, PA, USA

**Keywords:** colon, tight junction, cancer, cancer prognosis, inflammatory bowel disease, claudin, occludin

## Abstract

Colorectal cancer (CRC) is one of the most common cancers in the western world. Early screening and detection could be highly preventative and therefore reduce mortality. Tight junctions (TJ) are well known for their function in controlling paracellular traffic of ions and molecules. It has become increasingly evident that TJs play a crucial role in maintaining cell-cell integrity, and the loss of cell junctional sealing could involve itself in the processes of carcinoma and cancer metastasis. If correlations between altered TJ proteins and CRC presence or invasiveness could be established, they may serve as important markers and guidelines for prophylactic and prognostic purposes, along with other screening methods. This review will present recent data from clinical and animal studies showing how altered TJ protein expression is a feature of certain CRCs. The up-regulation of claudin-1 in many CRCs is especially noteworthy. The focus of this article is simply on the association – however imperfect – between CRC and the major TJ transmembrane barrier proteins, namely claudins and occludin. Any causal relationship between TJ protein change and neoplasia remains conclusively unproven at present.

## INTRODUCTION

Colorectal cancer (CRC) is the third most common cancer, with 150,000 cases diagnosed annually[1]. Mortality from CRC is highly preventable through early detection by screening programs and removal of precancerous adenomatous polyps. Guidelines recommend screening for the average-risk individual to begin at the age of 50. Earlier screening is recommended for patients at higher risk[2]. Colonoscopy is considered to be the "gold standard" of CRC screening, yet missed detection of lesions is possible[3]. Other tests are available and are endorsed by many medical societies, yet no test, including colonoscopy, is 100% sensitive in detecting early CRC[2]. These tests include stool test with guaiac, fecal immunohistochemical testing, stool DNA testing, flexible sigmoidoscopy, and computed tomography (CT) colonography[2]. Despite the availability of multiple tests effective for the early recognition of CRC, approximately 50,000 deaths related to CRC still occur each year, making it the second most common cause of cancer-related death in the U.S.[1]. This may relate to the fact that only about 50% of the U.S. population is enrolled in a CRC screening program[4]. Still, 50,000 annual deaths against a backdrop of 150,000 new cases annually suggest that there is a need to improve screening and detection rates. Newer strategies, such as increasing the numbers being screened for CRC or developing more sensitive testing modalities, are required to ensure that preventable deaths are avoided. Tight junctional proteins may represent possible markers for such testing.

Tight junctions (TJs) are the gasket-like seals that encircle each columnar epithelial cell around its apical pole (Fig. 1). They serve two roles: (1) they help to maintain cell polarity by physically separating the apical and the basolateral membrane domains and (2) they prevent free interchange of substances by diffusion along the paracellular pathway between the luminal and antiluminal tissue fluid compartments. Claudins and occludin are the most prominent protein members of the TJs. They span the cell membrane and interdigitate with the same proteins from the neighboring cells. These associations create a barrier and confer each barrier's particular, unique permeability characteristics. The uniqueness is helped by the existence of 24 distinct claudin genes and proteins, which are expressed in different epithelial tissues in different permutations. These structural and functional aspects of TJs are the subjects of frequent reviews[5,6,7].

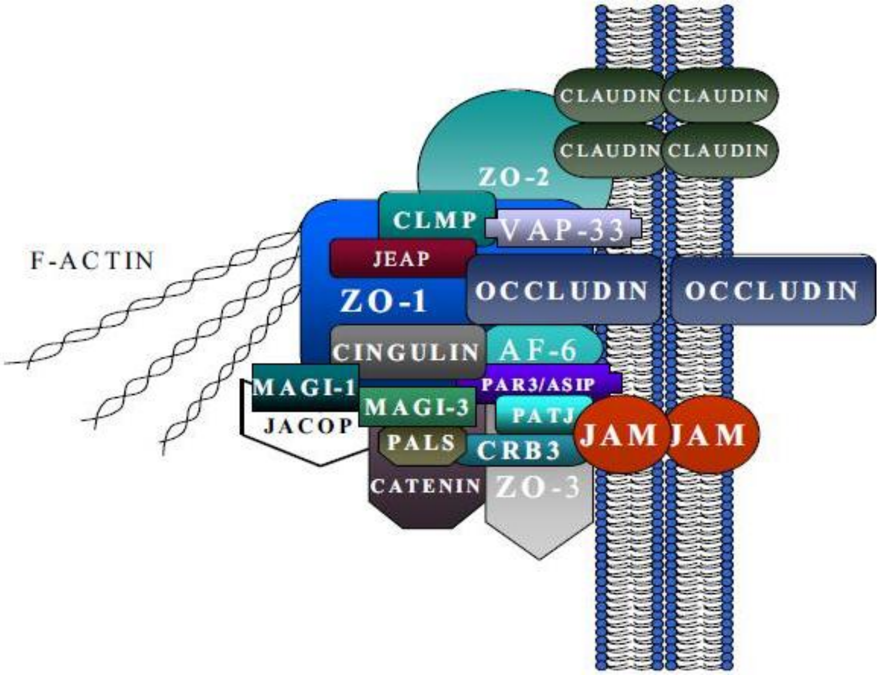

**FIGURE 1.** Core TJ structure composed of transmembrane proteins, TJ-associated proteins, and scaffolding proteins that fuse the lateral membrane of adjacent cells, restricting solute movement and maintaining polarity. (Reprinted from Maher et al. [2008] *Crit. Rev. Ther. Drug Carrier Syst*. **25(2),** 117–168. Reproduced with kind permission from Begell House, Inc. Publishers.)

The majority of cancers appear to arise out of epithelial cells and tissues. In cancer and other disease states, alterations in TJs often result in increased paracellular leakage[8]. This has several clinical implications. Novel diagnostic and prognosticating methods might be developed by focusing on changes in claudin expression and claudin post-translational modification in cancers. For example, sharp up-regulation of claudin-19 in a tumor biopsy may serve to indicate that this specific tumor is highly aggressive and may or may not respond to planned chemotherapy. In this sense, claudins serve merely as another "tumor marker", but their large family diversity and tissue specificity makes such typing increasingly attractive. As stated above, TJs have a very specific function, and abrogation of such function in epithelial neoplasia creates pathophysiologic clues and research opportunities of its own.

Altered TJ structure and increased TJ leakiness in cancer has been noted for quite some time[9,10,11,12,13]. The leak has implications for both cancer progression and cancer detection, and possibly therapy as well[9]. The measurement of increased probe leakage from tissue fluid compartment A to tissue fluid compartment B could theoretically serve as a test to identify neoplasia. Induction of leakage in TJs appears to be an early event in neoplastic progression in the colon and other epithelial tissues[8,10,11]. For example, our group has speculated whether TJ seals in Barrett's esophagus (metaplastic precursor to esophageal adenocarcinoma) are sufficiently leaky to serve as a clinical detection mechanism[12]. It is quite possible that elevated serum prostate-specific antigen (PSA) levels in prostate cancer are in fact due to paracellular leakage of PSA, a luminally secreted protease, from the prostatic lumen into the bloodstream[13].

It is interesting to consider whether such a leak contributes to the progression of cancer. Leakage of certain substances from a luminal compartment into the interstitium and vasculature (or vice versa) may play a role in tumorigenesis. There are likely two main mechanisms that are associated with leakage. An interplay between inflammation and cancer is suspected by many. Antigens, toxins, or other substances at high levels in the lumen that cross a barrier may trigger an inflammatory response in the interstitium. Due to the barrier (and specifically TJ) disruptive effects of proinflammatory cytokines[14], leakiness will beget further leakiness and inflammation. The second scenario concerns growth factors. These proteins are often at very high levels in luminal fluids. An excellent example is epidermal growth factor (EGF) in saliva, where the concentration can be 5,000 times greater than the level of EGF in blood[15]. Any chronically open paracellular leak in the mucosa of the upper or lower gastrointestinal tract may allow luminal EGF to cross the epithelial barrier and trigger mitogenic and cell motility responses in the epithelia or lamina propria after interacting with basolaterally situated EGF receptors[16]. In addition, as pointed out by Singh et al. in their own very recent, excellent review of TJs and cancer, TJ proteins are part of a complex composed in part of signaling proteins, which themselves may be involved in neoplasia[17]. The occurrence of claudin-1 in the nucleus of colon cancer cells may likewise play a signaling role in cancer development

This present review is, regrettably, mostly a mere cataloging of TJ protein changes occurring in colon cancer. There are studies that discuss the ability of transfected occludin to "roll back" the transformed phenotype[18] or the transfection of claudin-1 to enhance epithelial to mesenchymal transition (EMT) in colon cancer cell lines[19]. There are the longstanding observations by the Mullin group that protein kinase C–mediated induction of TJ leakiness and EMT in fact means that these are a direct effect of tumor-promoting chemicals[20,21]. There is also the model first put forward by Mullin of growth factor ligand/receptor compartmentation that breaks down in cancer by TJ disruption[14]. But all of these fall short of a true "smoking gun" that links TJ disruption and neoplastic progression in a causal fashion.

In summary, there are several reasons why changes in TJs during neoplasia merit attention. Although we have only an incomplete profile of the TJ protein changes accompanying neoplasia at the present time, a more complete picture holds not only diagnostic/prognostic value, but mechanistic insights as well. In the case of colon adenocarcinoma, certain basic patterns are already beginning to emerge.

## CLAUDIN-1

### Overall Expression in CRC

The published literature shows a strong association between colon neoplasia and increased claudin-1 expression. It is possible, though, that an aggressive subclass of tumors shows little, if any, claudin-1 activity.

In a study focusing on the involvement of claudin-1 in human colorectal tumorigenesis, the expression of claudin-1 was noted to be increased in CRC[22]. Human specimens from colon cancer surgeries were examined, with adjacent normal tissue serving as the control for each specimen. All 16 specimens demonstrated increased expression of the claudin-1 gene quantified by real-time polymerase chain reaction (RT-PCR). Immunohistochemical staining was used to determine the cellular location of increased protein expression of claudin-1, which was found to be most concentrated at the cell-cell boundaries and in the cytoplasm of cancer cells. Also at the protein level, Western blot analysis in a separate study showed a 5.7-fold increased expression of claudin-1 in all 12 human colon adenocarcinoma samples compared with adjacent normal colorectal mucosa[23]. The increase in claudin-1 expression was noted in both cytosolic and membrane/cytoskeleton–associated fractions. A similar observation was reported using CRC patient tissues that had undergone elective curative surgery[24]. They also found that both claudin-1 gene transcription and protein expression were up-regulated in the majority of CRC cases.

In an attempt to further develop the relationship among claudin-1 expression, localization, and colon cancer, Dhawan and colleagues found increased expression and frequent nuclear localization of claudin-1 in human primary colon carcinoma[19]. Up-regulation of claudin-1 may contribute to cellular changes during malignancy, including loss of cell polarity, abnormal cellular organization, and a decreased cell differentiation. These changes result in a compromised TJ barrier function, which may in turn increase the access to nutrients and signaling peptides[14], increase cell proliferation[25], and increase motility and metastasis[26] in states of malignancy. This study examined sections of human colon specimens comparing normal mucosa to primary carcinoma and to metastatic cancer tissue of the liver and lymphatics, which originated from the colon. The tissues were analyzed via immunohistochemistry using a polyclonal rabbit anti–claudin-1 antibody. Colon carcinomas and metastatic lesions showed an enhanced claudin-1 staining compared with normal mucosa, localized largely in the nucleus and cytoplasm. There was more consistently intense immunoreactivity from a statistically higher percentage of cells for primary carcinoma and in metastatic lesions compared with normal mucosa. Immunoblotting also showed that the level of claudin-1 expression was higher in primary human colon tumor lysates and lysates from metastatic lesions than in adjacent normal colon tissue. This study further investigated the relationship between claudin-1 as a target of the *adenomatous polyposis coli* (APC)/β-catenin pathway. In the past, mutation of the APC gene, and thus β-catenin activation and nuclear translocation, has been reported to occur in a majority of human CRC[27]. Nuclear localization of several intracellular cell-junction proteins (β-catenin, ZO-1, ZO-2) has been known to be correlated with oncogenic transformation and cell proliferation[28,29]. Findings by Dhawan et al. further support the opinion that nuclear and cytoplasmic mislocalization of claudin-1 is a common event in tumorigenesis and is associated with loss of APC tumor-suppressor function with activation of nuclear β-catenin[19].

## Regulation of Claudin-1 by Intracellular Mediators

In order to confirm that claudin-1 is indeed a target of the APC/β-catenin pathway, immunohistochemical analysis for claudin-1 was carried out in precancerous intestinal adenomas from ApcMin/+ mice. Claudin-1 protein levels were found to be highly elevated, with expression mislocalized from the membrane to the nucleus and cytoplasm. This is in contrast to the normal intestinal epithelium of heterozygous ApcMin/+ mice that express wild-type APC, where claudin-1 immunoreactivity was detected almost exclusively in the cell membrane with only low activity in the cytoplasm. These observations support the conclusion that regulation of claudin-1 expression is an important role of the APC gene, and the mutation of APC would therefore result in abnormal claudin-1 expression in the process of tumorigenesis[19].

Further investigations into the relationship between claudin-1 expression and CRC have focused on Smad4, a tumor-suppressor, intracellular signal transduction component of the transforming growth factor-β (TGF-β) family of cytokines. Loss of Smad4 function, either due to genetic mutation or loss of expression, has been reported in a significant proportion of colon and pancreatic cancers[30,31,32].

Frequent loss of Smad4 expression in tumors that have acquired invasive and metastatic phenotype strongly implicates the tumor-suppressor function of Smad4[33]. A study published in 2007 examined tissue samples from six patients with CRC to try to better understand the relationship between claudin-1 and Smad4[34]. Immunoblotting assays showed that claudin-1 expression was minimal in normal colon tissues and increased in tumors. Smad4 expression was, however, abundant in normal samples, while its expression was often decreased or was undetectable in tumors. Both increased claudin-1 expression, and decreased or absent Smad4 expression, occurred in tandem in four out of six CRC samples, suggesting a possible inverse relationship between the two. Similarly, in several colon cell lines, claudin-1 expression was only detected among cell lines that did not express Smad4. These findings together led the authors to suggest that the inverse relationship between Smad4 and claudin-1 in CRC may represent regulation of claudin-1 by the Smad4 tumor-suppressive function[34].

Smad7, like Smad4, is an intracellular signal mediator involved in the TGF-β signaling cascade. Overexpression of Smad7 in colon adenocarcinoma–derived (FET) cells has been shown to induce tumorigenicity by blocking TGF-β–induced growth inhibition and apoptosis[35]. The relationship among hepatic metastasis of CRC in mice, junctional proteins, and Smad7, was explored further by the same group[36]. In an experimental model of colon cancer liver metastasis, FET cells that express Smad7 were injected into the mouse spleen. Diffuse liver metastases were revealed 33 days after the injection, which were then analyzed via immunohistochemistry, RT-PCR, and Western blot analysis for expression of a host of junctional proteins and proteins involved in the TGF-β signaling pathway. PCR from tissue derived from liver metastases demonstrated that cells expressing Smad7 had migrated to the liver. Both Western blot and immunohistochemistry demonstrated strongly elevated levels of claudin-1 and -4 in Smad7-mediated liver metastases. The authors concluded that blockade of the TGF-β/Smad pathway in colon cancer cells induces metastasis, supporting an important role of Smad signaling in inhibiting colon cancer metastasis with claudin-1 as a potential target of the Smad signaling pathways.

## Claudin-1 Expression in CRC Associated with Inflammatory Bowel Disease (IBD)

TJ activity has also been examined in a subset of patients with IBD, which includes ulcerative colitis (UC) and Crohn's disease (CD). Since these patients have a higher lifetime risk of CRC, they represent an important subgroup for analysis at the microscopic level[37,38,39]. This may also have implications in regards to progression from IBD to CRC, as recent data show histological inflammation as a risk factor towards CRC in patients with UC[40]. This may also be true in CD, as the characteristics and prognosis of CRC in CD also have been shown to be similar to those for CRC in UC[41].

In a study scrutinizing the relationship of IBD and early onset of neoplasia, the authors examined 16 biopsy and colectomy specimens from 15 patients with IBD, 12 who had UC and 3 who had CD[38]. Semi-quantitative immunohistochemical staining of human biopsies was used to assess the expression of TJ proteins, including claudin-1 in four different tissue categories: active IBD; IBD-associated dysplasia; acute, self-limited colitis (ASLC); and sporadic adenomas. The active, but not the inactive IBD group, demonstrated an increased claudin-1 expression compared to controls, which was statistically significantly elevated within epithelia adjacent to lymphoid aggregates. In addition, active bowel inflammation was also found to be associated with a greater localization of claudin-1 to lateral membranes. In the IBD-associated dysplasia group, claudin-1 expression was similarly elevated in comparison to nondysplastic IBD tissue. In the ASLC group, no significant elevations in claudin-1 expression were seen, whereas in the sporadic adenoma group, increased claudin-1 expression was observed in over 90% of the specimens, with localization of this expression to the lateral membranes of dysplastic colonocytes. Similar elevation of claudin-1 expression was also seen in three cases of invasive carcinoma associated with IBD[38].

A recent study assessed claudin-1 expression in human colon specimens of patients with CRC and concomitant UC using immunofluorescence techniques[37]. Carcinoma specimens were compared to specimens containing both intraepithelial neoplasia as well as normal mucosa from the same patient. Using a semi-quantitative intensity-scoring system, both crypts and surface mucosa of CRC specimens demonstrated a significantly elevated expression of claudin-1 compared with both intraepithelial neoplasia and normal mucosa specimens ($p < 0.05$). These data support a concept that both sporadic and UC-associated carcinoma develop along similar TJ protein pathways.

## Claudin-1 Expression and Clinicopathological Correlation: Prognostic Potential

Since elevated claudin-1 expression in CRC compared to controls has been well documented, several studies have attempted to correlate levels of claudin-1 expression with tumor grade and stage. One of the first of these studies demonstrated an inverse relationship[42]. The authors explored potential prognostic implications of claudin-1 expression in tumor-node metastic stage II CRC via examination of four different TJ proteins, including claudin-1. CRC surgical specimens (n = 129) were analyzed by tissue microarray technology followed by immunohistochemical staining for TJ proteins. In general, both the tissue array pattern and quantification of claudin-1 were examined in comparison to normal colon mucosa. In terms of pattern of staining, claudin-1 demonstrated a circumferential membranous staining pattern in both normal mucosa as well as in CRC. A more fragmented and weaker circumferential pattern of staining was mostly observed in poorly differentiated tumors. As a function of prognosis on the level of the overall quantification of claudin-1 protein, 75% of the tumors exhibited elevated or equal levels of claudin-1 compared to those seen in the normal colon mucosa. Despite the near unanimous evidence of elevated levels of claudin-1 in CRC, a paradoxical relationship was also noticed in this study: *absent or low* claudin-1 expression in carcinoma compared to controls correlated with poor differentiation and, perhaps more notably, strongly correlated with disease recurrence and poor patient survival. In terms of prognostic implications, the authors concluded that an *inverse* correlation existed between levels of claudin-1 expression and tumor grade. Ultimately the authors suggested that low or absent claudin-1 levels were a stronger predictor of recurrence and mortality than either lymphovascular invasion or grade[42]. However, some follow-up studies have not successfully replicated these findings, with a wide variety of results ranging from a direct relationship between claudin-1 level and tumor stage to no correlation whatsoever between the two[23,24].

Using CRC colectomy tissues, Grone et al. were unable to detect a statistically significant correlation between tumor stage and claudin-1 gene expression, or between clinicopathologic parameters and claudin-1 protein staining[23]. They also suggested that this may be due to the heterogeneous sample pools comprising all stages of colon and rectum cancer. A recent publication by Huo and colleagues demonstrated a correlation between the mRNA level of claudin-1 and tumor depth within sporadic CRC surgical specimens in Japan[24]. The mRNA levels of claudin-1 were elevated in more advanced cancer cases (penetration was deeper than muscularis propria) than earlier cancers (limited within mucosa and submucosa) ($p < 0.01$). It was also higher in the distal colon than in the proximal ($p < 0.05$), which may be due to the fact that the former site has a higher cancer incidence rate. Moreover, no associations were noted regarding age, gender, tumor size, lymph node metastasis, and carcinoembryonic antigen (CEA) level in this study[24].

In summary, claudin-1 levels in colon cancer may provide one of the more promising claudin cancer markers, referring here specifically to claudin-1 elevation in many CRCs. However, the correlations are not perfect and more work clearly needs to be done regarding claudin-1 in differentiated vs. relatively undifferentiated CRCs.

## CLAUDIN-2

## Overall Expression in CRC

Like claudin-1, claudin-2 is a transmembrane protein that has also been identified as an integral component of TJ strands[43]. Expression of claudin-2 has also been measured in human CRC

tissues[44,45], in mice[46], and in patients both with and without a history of IBD[38]. So far, the findings have been mixed. Some studies show no change[37], while others show a decrease[47], and still others found an increase in claudin-2 expression in CRC/IBD compared to normal colon tissue[38,45].

A Finland-based study sought to explore the expression of claudin-2 in various states of malignancy, including colon cancer[48]. Using 12 CRC tissue specimens, analyses for claudin-2 expression via immunohistochemistry were undertaken. More than half of the samples (60%) tested positive for claudin-2. However, this study was limited by a small sample size, limited methodology, and lack of a control arm.

The previously mentioned study by Huo et al. also examined the expression of claudin-2 mRNA in 41 human CRC tissue specimens compared to normal colon mucosa[24]. Data from quantitative RT-PCR analysis demonstrated a 25-fold *increase* in claudin-2 mRNA expression in CRC tissues compared to normal colon mucosa. Yet, there was no further information on the protein expression in CRC tissues.

In order to determine whether claudin-2 expression is specific for cancer, human tissues were utilized to evaluate the expression of claudin-2 in normal vs. malignant conditions in a separate study[45]. A small number of CRC samples (n = 9) was used for RT-PCR analysis, while 99 human CRC specimens were examined via immunohistochemistry with a healthy colon specimen serving as the control. Data from RT-PCR demonstrated *no* expression of claudin-2 whatsoever in the healthy colon tissue. In contrast, five of nine CRC specimens expressed claudin-2 at significantly high levels (more than five arbitrary units). Immunohistochemical staining revealed claudin-2 expression in cell membranes in 25 of 99 CRC specimens, compared to *no* staining identified in normal colon tissue. Overall, these results show an increased expression of claudin-2 in CRC compared to normal colon tissue.

On the contrary, in 2009, Hahn-Stromberg's team examined 33 human CRC resection specimens in an attempt to quantify claudin-2 expression in CRC vs. normal colon tissue[47]. They also examined the correlation of the expression of several junctional proteins, including claudin-2, with tumor location, stage, lymph node involvement, and degree of differentiation. First, immunohistochemistry was performed to quantify claudin-2 expression in the two groups. All 33 normal colon samples demonstrated some claudin-2 expression in 80–100% of the cell population. In comparison, 18 of 33 tumor samples showed claudin-2 expression in only 10–50% of cells, and 14 of 33 CRC samples showed claudin-2 expression in 50–80% of cells. Only 1 of 33 CRC specimens showed 80–100% claudin-2 expression in the cell pool stained. These findings demonstrate a *decreased* expression of claudin-2 in CRC tissues compared to normal colon sections. Second, the use of computer-assisted image analysis allowed the analysis of the 33 human CRC specimens to determine whether a relationship existed between claudin-2 expression and tumor volume or tumor growth pattern. However, this study found no correlation[47].

## Expression in CRC Associated with IBD

The aforementioned 2009 study by Mees et al. used immunofluorescence and an intensity-scoring system to assess claudin expression, including claudin-2, in human colon specimens of patients with CRC and concomitant UC[37]. Carcinoma specimens were compared to specimens containing both intraepithelial neoplasia as well as normal mucosa from the same patient. Regarding claudin-2, there was no difference in the expression among CRC, intraepithelial neoplasia, and normal mucosa samples.

In addition to claudin-1 mentioned previously, Weber et al. explored the relationship between claudin-2 expression and various states of IBD. Semi-quantitative immunohistochemical staining of human biopsies was used to assess expression of claudin-2 in different tissue categories: active IBD, IBD-associated dysplasia, and sporadic adenomas. The findings of claudin-2 were similar to those of claudin-1; claudin-2 was found to be increased in active IBD, IBD-associated dysplasia, and in sporadic adenomas[38].

In summary, there is evidence that claudin-2 expression is low in the normal colon and elevated in CRC/IBD, but there is by no means universal agreement on this. More studies are clearly needed. However, it should be pointed out that in findings made by immunofluorescence or immunocytochemical detection of claudins using fixed, histological sections of tissue, it would be useful to see if confirmation of findings could be obtained by Western immunoblot methods. A drawback of immunocytochemistry and immunofluorescence, especially with polyclonal antibodies to claudins, is that fluorescence or colored stain might indicate the presence of the desired claudin, *or* it might reflect the presence of an unrelated protein being recognized by the antisera. Some antisera are “cleaner” than others in this regard.

## CLAUDIN-3 AND CLAUDIN-4

### Overall Expression in CRC vs. Normal Colon Tissues

Increased levels of claudin-3 and -4 expression have been reported in the majority of ovarian carcinomas, shown to be closely correlated to the grade of malignancy[49]. Claudin-4 on its own has also been found to demonstrate strong immunostaining in primary and metastatic pancreatic cancers[50]. With this in mind, several authors have examined the expression of claudin-3 and -4 in CRC in comparison to normal colon tissues. Overall, the data show a slightly increased expression of claudin-3 and -4 in CRC compared to normal colon tissue, albeit with a much less robust significance than those found for claudin-1.

In one study, paired colorectal adenocarcinoma tissues with normal mucosa in 12 human resection specimens were examined in an effort to scrutinize the role of altered expression of TJ protein in colorectal adenocarcinoma[51]. Western blot analysis was undertaken to quantify protein expression in normal vs. neoplastic tissue. Claudin-3 and -4 were increased in cancer tissues by an average of 1.5- and 2.4-fold, respectively. The subcellular location of claudin-3 and -4 was also investigated via quantification of cytosolic and membrane/cytoskeleton fractions. Both claudin-3 and -4 were found to be increased in the insoluble fraction by 1.5- and 2.5-fold, respectively, suggesting an elevation in the membrane/cytoskeleton–associated fraction of tumor tissues. When the electron-dense dye ruthenium red was utilized to assess the barrier function of the epithelia, the tumor tissues demonstrated a higher level of leakiness (increased paracellular permeability to ruthenium red dye) compared to the normal ones. The disturbed claudin expression in the tumors may have contributed to the leakiness. One might think that “more” of a given claudin is a “good thing” in terms of barrier enhancement, but one cannot be sure that this is true and may be a vast oversimplification of how these barriers work. The original homo- and heterotypic interaction models put forward by Furuse et al. would, in fact, suggest that barrier impairment can result from either “less” of a certain claudin or “more” of that claudin[52]. We would speculate that it is the proportion of claudins in a junction that is critical, and neoplasia-derived changes in proportionality of claudins are injurious to the barrier function of a junctional seal because of disturbances in homo- and heterotypic interactions.

At the level of gene expression, a study by Hewitt et al. was carried out to explore the patterns of 21 different claudins in normal vs. neoplastic tissue[44]. Using the public Serial Analysis of Gene Expression (SAGE) database, the authors developed gene-specific primers for 13 different claudin genes. RT-PCR was then used to examine gene expression of claudin genes in normal vs. neoplastic tissue in a variety of organs, including colon. Claudin-3 and -4 were found to be present in normal colon tissue as well as in neoplastic colon tissue, with elevated transcription in tumor specimens compared to normal tissues.

In addition to claudin -1 and -2, the previously mentioned 2009 study by Huo et al. also examined the expression of claudin-3 and -4 in 41 human CRC tissue specimens compared to normal colon mucosa[24]. However, unlike the 25-fold increase seen in claudin-1 and -2 mRNA expressions in CRC vs. normal colon tissue, RT-PCR data demonstrated only a two- to threefold increase in claudin-3 and -4 mRNA expression, respectively. This increase was also not deemed to be of statistical significance.

### Expression in CRC Associated with IBD

The data regarding altered levels of claudin-4 expression in CRC associated with IBD compared to controls is a mixed story. The aforementioned study by Weber et al. explored claudin-4 expression in 16

biopsy and colectomy specimens from 15 patients with IBD; 12 with UC and 3 with CD. As previously described, immunohistochemical analysis was performed in specimens with various grades of inflammatory and neoplastic activity. Claudin-4 expression was enhanced in tissues with grade 2 inflammation compared to controls, with intense membranous staining present ($p < 0.05$). However, this trend was *not* seen with grade 3 inflammatory activity, where claudin-4 expression was variable and not significantly increased vs. controls. Furthermore, claudin-4 expression in adenomas or carcinomas was no different than that seen in controls[38].

In a study led by Mees et al., the researchers examined 16 colectomy specimens from patients with UC[37]. In addition to other TJ proteins, claudin-4 activity was examined in CRC specimens vs. adjacent mucosal controls. Similar to claudin-1, claudin-4 was found to be significantly elevated in the CRC group compared to both intraepithelial neoplasia and controls using an intensity-scoring system ($p < 0.05$).

## Expression as a Function of Prognosis

Determining whether claudin-4 levels in CRC tissues can be correlated with tumor stage or prognosis remains to be seen. A large study published in 2005 showed that, as was true for claudin-1, an association existed between lower levels of claudin-4 expression and more advanced stages of CRC[42]. This finding was replicated a year later by the Morin group. It also showed that decreased claudin-4 levels were present in more poorly differentiated CRC specimens compared to well-differentiated specimens[44]. Higher rates of tumor metastasis were also found to be correlated with lower levels of claudin-4 expression in the same study. However, without a clear correlation between overall claudin-4 expressions in CRC samples compared to controls, the implications of this finding remain unclear.

In addition to claudin-1, Resnick et al. also examined claudin-4 in 129 CRC surgical specimens by tissue microarray technology followed by TJ protein immunohistochemical stainings, as a function of tumor stage and grade[42]. Absent or low claudin-4 expression was observed in 55 of 129 (42%) samples compared with normal or elevated claudin-4 expression in the rest (58%) of the samples. These data made drawing conclusions regarding claudin-4 expression in CRC quite difficult. The loss of claudin-4 expression was not associated with differentiation or prognosis in this study, although claudin-4 levels have been shown to be up-regulated in other cancer types[49,50,53,54]. It is not clear why, in the same study, reduced claudin-1, rather than claudin-4, expression is significantly correlated with poor prognosis with the current knowledge; however, this finding does implicate that the down-regulation of certain claudin members has greater impact on cancer aggression than some others.

Ueda and colleagues aimed to explore the significance of claudin-4 in CRC compared to normal human colon tissues, and to correlate its association with clinicopathologic factors[29]. Using human tissue from 129 CRC and 44 metastatic lesions to lymph nodes, liver, lung, or wide dissemination, immunohistochemistry was performed to examine the level of claudin-4 expression. Claudin-4 immunoreactivity was found in normal colorectal surface and crypt epithelia in the region of the intracellular membrane. In comparison, in CRC, claudin-4 immunoreactivity tended to be preserved in well-differentiated adenocarcinomas, but was significantly decreased in both moderately and poorly differentiated adenocarcinomas ($p = 0.00013$). Moreover, the expression was heterogeneous, i.e., strong immunoreactivity of claudin-4 was detected on the surface of CRC tissue, while decreased claudin-4 immunoreactivity was noted at the invasive front and at regions of vessel infiltration. Furthermore, decreased protein expression of claudin-4 was significantly correlated with depth of invasion ($p < 0.0001$), invasive pattern ($p < 0.\ 0001$), lymphatic vessel invasion ($p < 0.0001$), and metastases ($p < 0.01$). These data indicated an inverse relationship between claudin-4 expression and metastatic lesions of CRC tissues.

This study also explored the relationship between immunoreactivity of claudin-4 and recurrence after surgical resection in 40 cases of stage II CRC, to determine the extent that claudin-4 expression was correlated with prognosis independent of clinical stage. Study of local recurrence in stage II CRC using multivariate Cox analysis revealed that cases with decreased claudin-4 expression tended to have more

(but not yet statistically significant) recurrent tumors than those with high histological grade or vessel invasion[29].

In summary, the majority – but not all – studies of claudin-4 transcription and expression in CRC suggest that decreased claudin-4 levels correspond with CRC per se and the aggressiveness of the cancer. Again, as with claudin-2, we speculate about the specificity of individual anti–claudin-4 antibodies in individual studies and their accurate indication of claudin-4 abundance, especially in immunohistochemistry.

## CLAUDIN-7

In addition to being a part of the TJ, claudin-7 has also been observed in the basolateral membrane and/or in cytoplasmic vesicles[55,56,57,58]. Down-regulation of claudin-7 through hypermethylation of the gene promoter region has been reported as a role player in the pathogenesis of breast cancer cell lines[59]. Claudin-7 has been studied in squamous cell cancer of the esophagus, where its down-regulation has been shown to lead to increased invasiveness of tumors[60]. Conversely, the up-regulation of claudin-7 has been associated with the tumorigenesis process in malignancies in the kidney[61] and stomach[62]. In relation to CRC, one study published in 2008 examined the role of claudin-7 in CRC tumorigenesis[46]. Specimens of colon tumors were matched with controls consisting of normal colon mucosa in 12 patients. Immunohistochemistry and immunofluorescence staining were used to examine claudin-7 expression in each sample. In normal colon epithelium, claudin-7 was found to be expressed throughout the crypts, but the intensity and nature of staining varied from the base of the crypt to the surface epithelium; namely, cells at the surface displayed a very strong claudin-7 expression compared to cells at the crypt base. In comparison, in CRC samples, claudin-7 was found to be strongly overexpressed throughout. Furthermore, based on the finding that transcription factor Sox-9–deficient mice have shown a very strong overexpression of claudin-7[63], the authors treated BALB/c nude mice with CRC cell lines (HT-29Cl.16E/Sox-9 cells). The majority of these mice (five out of six) were found to have large colon tumor xenografts 40 days after the initial injection, whereas none was found in control animals. These findings support a role for overexpression of claudin-7 in CRC tumorigenesis[46].

In a previously mentioned gene expression study by Hewitt et al., researchers explored the patterns of claudin-7 in normal vs. neoplastic tissue[44]. RT-PCR with gene-specific primers for claudin-7 allowed the authors to report that claudin-7 showed elevated transcription in CRC compared to normal colon tissue.

There has been a more recent suggestion that claudin-7 does not act independently, but instead is part of a complex that leads to CRC. Metastasizing gastrointestinal tumors of rats have been shown to frequently express a complex of four molecules that may promote tumor progression[64]; namely, the tetraspanin D6.1A (human: CO-029, which has been associated with a poor prognosis in gastrointestinal cancers [50]), EpCAM (a panepithelial homophillic cell-cell adhesion molecule that has been shown to be up-regulated in CRC[65]), claudin-7, and the tumor marker CD44 variant isoform v6 (CD44v6)[52,61]. Inspired by those findings, Kuhn and team followed up these findings using human tissue[64]. Using antibodies to the complex of proteins above, immunohistochemistry was performed in primary colorectal cell tissue (n = 104) and liver metastases (n = 66), and compared with normal colon and liver tissue as controls. Moderate to strong staining of EpCAM, claudin-7, CO-029, and CD44v6 was seen in 73, 53, 81, and 36% of CRC vs. 10, 3, 1, and 5% of corresponding tumor-free colorectal tissues, respectively. The same antibodies stained 74, 38, 75, and 20% of liver metastases, and 8, 0, 6, and 0% of tumor-free liver tissue from the same patients, respectively. In terms of correlation with clinicopathologic prognosis, expression of the four molecules did not correlate with tumor staging and grading. However, the authors suggested that coexpression of the four molecules together inversely correlated with disease-free survival[64].

## CLAUDIN-8

Claudin-8 has also been examined in CRC tissues, albeit in a more limited fashion. In the aforementioned 2007 study by Grone et al., scientists used human tissue from 30 patients with CRC to hybridize gene chips and to analyze the expression of genes encoding TJ proteins, including claudin-8 in normal vs. neoplastic tissue[23]. On the level of RNA, claudin-8 was significantly down-regulated in CRC tissue in 27 of 30 patient samples. Moreover, in more than 75% of all patients, claudin-8 showed more than a 10-fold down-regulation in tumor samples. However, there was no correlation linked between tumor stage and claudin-8 gene expression. Western blot analysis of tissue samples from eight patients with CRC compared to matched controls was also performed for claudin-8 protein expression. A strong claudin-8 expression was demonstrated in all normal tissues, while in contrast to RNA level, tumor tissues only showed a slightly reduced signal intensity that did not reach statistical significance[23].

## CLAUDIN-12

Similar to claudin-8, claudin-12 is another TJ protein that seems to be involved in CRC development, but has not been investigated as intensively as other previously mentioned members of the claudin family. Using CRC surgical biopsies, Grone et al. observed an overexpression of claudin-12 on the RNA level in over 40% of patients[23]. Quantification of protein levels also revealed a trend towards up-regulation; however, this trend was not deemed to be significantly different. So far, this is the only report on this claudin and it needs further analysis.

## OCCLUDIN

### Expression and CRC

Occludin was one of the first identified proteins in the TJ, and is considered an integral part of the TJ regarding structure and possible signaling[66]. Occludin, larger than the typical 20–25 kDa claudins, is a 65-kDa membrane protein with four transmembrane domains forming two extracellular loops and a long cytoplasmic tail[67]. It has also been shown to be linked with apoptotic machinery involving mitogen-activated protein kinase and Akt signaling pathways in murine hepatocytes. The signaling properties and its role in the TJ have warranted investigation of the expression of occludin in carcinogenesis. As previously described, impairment of proteins of the TJ has been implicated in carcinogenesis, and genes having an oncogenic nature have been shown to impair TJ integrity[18,68,69]. The expression of occludin has been shown to be down-regulated with the progression of human endometrial carcinoma[70]. In human CRC tissues, existing data generally show a decreased expression in comparison with normal controls[47,69]. Further, this decrease has also shown a correlation with tumor grade; more advanced lesions show progressively *less* expression of occludin compared to less advanced lesions[42].

The first study examining occludin expression in human CRC tissues was published in 1997 by Kimura et al.[69]. Human colon adenocarcinoma specimens (n = 12) were studied with noncancerous adjacent mucosa serving as controls. Immunohistochemistry demonstrated a decreased expression of occludin in CRC vs. normal colonic epithelium. This trend tended to correlate with tumor grade. Normal epithelium showed occludin expression in 90–100% of cells in all 12 samples. In comparison, in eight samples of grades I and II CRC, occludin expression was present in only 50–90% of cells. In one sample of grade II CRC and two samples of grade III CRC, it was present in only 10–50% of cells. In one sample of grade III CRC, no occludin expression was able to be identified at all. Incidentally, however, the authors demonstrated a similar inverse relationship of occludin expression and histopathologic tumor grade in 19 samples of gastric adenocarcinoma.

In the previously mentioned study by Resnick et al., researchers also included occludin in the examination of TJ protein expression as a function of tumor grade in 129 human CRC specimens[42]. Immunohistochemistry was used to examine colon cancer tissue with normal colon mucosa serving as controls. Compared to normal mucosa, absent or low occludin expression was seen in 55 of 129 (42%) tumor specimens; 43 well-differentiated and 12 poorly differentiated. In comparison, 71 of 129 (55%) samples showed normal or elevated levels of occludin expression, 64 of which were in well-differentiated specimens compared to 7 poorly differentiated ones. An inverse, but yet statistically insignificant, relationship/trend between occludin expression and tumor grade was therefore identified; however, with this alone, one could not sufficiently draw a conclusion on occludin expression in CRC.

Nevertheless, still another study showed occludin expression in 33 human CRC specimens examined via immunohistochemistry to be decreased, although not statistically significant, compared to normal colon samples. Thirty-three normal tissue specimens showed occludin expression via immunohistochemistry in 80–100% of stained cells, while only 17 CRC specimens showed occludin expression in 80–100% of cells, and 16 other CRC samples showed expression present in only 50–80% of cells[47]. Some changes seen in claudin and occludin are summarized in Table 1.

**TABLE 1**
**Changes in TJ Protein Expression in Human CRC Samples**

| | Changes | Refs. |
|---|---|---|
| Claudin-1 | ↑ | [19,22,23,24,37,51] |
| Claudin-2 | No difference | [37] (mice) |
| | ↑ | [45] |
| | ↓ | [47] |
| Claudin-3 | ↑ | [37,44,51] |
| Claudin-4 | ↑ | [37,44,51] |
| Claudin-7 | ↑ | [44,46] |
| Claudin-8 | ↓ (RNA level) | [23] |
| Claudin-12 | ↑ | [23] |
| Occludin | ↓ | [47,69] |

## Expression in CRC Associated with IBD

In terms of occludin expression in IBD and IBD-related CRC, no real correlation has been demonstrated. Among other TJ proteins, the study carried out by Weber et al. also explored the occludin protein activity in 16 biopsy and colectomy specimens from 15 patients with IBD, where 12 patients had UC and 3 had CD[38]. Immunohistochemical analysis was performed in specimens with various grades of inflammatory and neoplastic activity. Occludin expression and localization in both active and inactive IBD were similar to that in control tissue. Similarly, occludin expression in adenomas or carcinomas was no different than that seen in controls. Likewise, Mees and colleagues examined 16 colectomy specimens from patients with UC and found no significant difference in occludin according to immunohistochemical intensity between CRC and adjacent healthy mucosal controls[37]. Although UC and CD are fully distinct diseases, it is noteworthy that the above results contrast starkly with those of Zeissig et al., which showed dramatic reduction of occludin levels in the mucosa of patients with active CD[71].

## JAM AND TRICELLULIN

Concerning junctional adhesion protein (JAM) and tricellulin, there have been very limited studies focused on their content in CRC. IBD conditions have been mimicked in a JAM-A knock-out mouse model[72]. Increased colonic intestinal permeability, claudin-10 and-15 expression, and inflammation were also observed as the result of the knock-out. Although this very important study demonstrated a complex role of JAM-A in maintaining colonic homeostasis, clinical tissue-based studies are needed to reveal the relationship between its expression and CRC. Similarly, the recently discovered TJ molecule tricellulin requires special attention in the field of CRC and IBD. The potentially unique role of tricellulin in serving as a site for macromolecular penetration across an epithelial barrier makes it perhaps the most attractive TJ protein to study in both IBD and CRC[73].

## CONCLUDING REMARKS

Cancer perennially seems able to defy any generalizations applied to it; for every trend, there is a strong exception. Correlations concerning TJ proteins with colon tumor vs. normal colon tissue, or with degree of colon cancer aggressiveness, fall well short of universality. Typically, studies showing up-regulation of claudin-X in colon cancer can be offset by studies showing no change or even down-regulation of a claudin family member. Again, given perhaps the inherent phenotypic malleability of neoplasia, this is not surprising. One claudin that seems to somewhat buck this haphazard expression tendency is claudin-1. Counterintuitively up-regulated in most colon cancer, the minority of colon adenocarcinomas in which it *is* sharply down-regulated associate very well with a high degree of invasiveness and a poor patient prognosis. Perhaps as research continues, other TJ proteins, for which there is as yet a small amount of data, may yet show as strong an association with clinical findings as does claudin-1. In any event, one truism to emerge concerning TJ proteins and colon cancer cells is that location is the key in more fields than simply real estate. A very strong correlation is that when a TJ protein is up-regulated in colon cancer, one needs to look for it in subcellular locations other than simply the junctional region, with cytosol being a favored location. It speaks to the fact that these proteins are likely performing functions in the cell other than simply the structural, barrier roles for which they are best known.

It is quite maddening (including to us, the authors) as one reads our review to encounter this infuriating inconsistency in the behavior of claudins in colon cancer (and cancer in general). However this inconsistency is certainly consistent with cancer. Cancers are not simply inconsistent from person-person or case-case, but, worse still, they are plastic and can modify with time within a single individual (case). One need only witness their ability to eventually modify their phenotype to escape the toxicity of specific chemotherapeutics. So we are really not surprised that universalities do not seemingly exist regarding claudin changes in colon cancer. And this may say something singularly noteworthy about the philosophical thrust of much biomedical research. *It may be overly molecular in this case.* Perhaps instead of looking for claudin "molecular signatures" in this disease (and the signaling pathways/transcriptional regulation responsible for these changes), researchers need to be a bit more phenomenological and descriptive in their approach. It is not a good step to take to attain funding, but it may more adroitly address key changes in epithelial neoplasia. We perhaps should "back away" and ask the simpler, broader (*and functional*) question as posed by Soler et al.[10]; namely, do TJs become uniformly *leaky* in cancer? The complexity of claudins (one can have a lot of permutations with a n = 24 family) may obfuscate attempts at a specific, uniform molecular change. But whether one particular claudin's abundance goes up or another specific claudin's abundance goes down in a specific neoplasia, the net result may much more uniformly be *leakiness*. This, perhaps, is where future research should focus – i.e., regardless of the exact form of the claudin change, is leakiness per se a signature of particular cancers?

## ACKNOWLEDGMENTS

This work was supported in part by a grant from the Sharpe-Strumia Foundation (J.M.M.).
